# The Effect of Structural Design on Mechanical Properties and Cellular Response of Additive Manufactured Titanium Scaffolds

**DOI:** 10.3390/ma5081336

**Published:** 2012-08-10

**Authors:** Jan Wieding, Anika Jonitz, Rainer Bader

**Affiliations:** Department of Orthopedics, University Medicine Rostock, Doberaner Strasse 142, Rostock 18057, Germany; E-Mails: anika.jonitz@med.uni-rostock.de (A.J.); rainer.bader@med.uni-rostock.de (R.B.)

**Keywords:** additive manufacturing, titanium, bone scaffold, mechanical properties, compressive strength, elastic modulus, *in vitro*, human osteoblasts

## Abstract

Restoration of segmental defects in long bones remains a challenging task in orthopedic surgery. Although autologous bone is still the ‘Gold Standard’ because of its high biocompatibility, it has nevertheless been associated with several disadvantages. Consequently, artificial materials, such as calcium phosphate and titanium, have been considered for the treatment of bone defects. In the present study, the mechanical properties of three different scaffold designs were investigated. The scaffolds were made of titanium alloy (Ti6Al4V), fabricated by means of an additive manufacturing process with defined pore geometry and porosities of approximately 70%. Two scaffolds exhibited rectangular struts, orientated in the direction of loading. The struts for the third scaffold were orientated diagonal to the load direction, and featured a circular cross-section. Material properties were calculated from stress-strain relationships under axial compression testing. *In vitro* cell testing was undertaken with human osteoblasts on scaffolds fabricated using the same manufacturing process. Although the scaffolds exhibited different strut geometry, the mechanical properties of ultimate compressive strength were similar (145–164 MPa) and in the range of human cortical bone. Test results for elastic modulus revealed values between 3.7 and 6.7 GPa. *In vitro* testing demonstrated proliferation and spreading of bone cells on the scaffold surface.

## 1. Introduction

Large segmental defects in long bones still represent a challenging task in orthopedic surgery. These defects can be caused by fracture, tumor or infection, with varying severity [1,2,3]. For large defects (critical size defects), regeneration cannot be accomplished by the patient’s body alone. Further assistance is needed, which is provided by filling the defect with bone scaffold materials.

Autologous material is still deemed the “Gold Standard” for treating this kind of defect, because of its high biocompatibility [4]. However, grafting of autologous material is associated with problems, which include donor site morbidity, limited availability and the necessity of a second surgery with further consequences for the patient [5,6]. Therefore, artificial materials, such as calcium phosphate and metals, have been investigated with regard to the treatment of bone defects, and are used with increasingly frequency [7,8]. Metallic materials like titanium and its alloys have performed particularly well in clinical applications, are commonly available, and can be manufactured in a wide range of scaffold designs. For sufficient bone ingrowth into the scaffold, cell biological, in addition to biomechanical, properties play a crucial role in the initial stability of the bone-implant composite system. For bone ingrowth, an open-porous structure and adequate pore sizes must be guaranteed [9].

Furthermore, mismatch of the mechanical properties, between scaffold and surrounding tissue, can lead to stress shielding around the scaffold and subsequently inhibit tissue ingrowth or cause implant loosening [10,11]. Therefore, the mechanical properties of the implants acting as scaffolds for bone ingrowth should be adapted to the mechanical properties of the surrounding tissue [12].

In addition to different materials, open-porous structures (*i.e.*, porosity) can reduce the mismatch between the scaffold and surrounding tissue. Porosity, pore size and interconnecting pores are also essential for sufficient bone ingrowth [13,14].

Porosity of the scaffolds can either be achieved by foam-like structures with irregular pore geometry and stochastic pore distribution with varying pore sizes [9,15,16], or by lattice structures with regular geometry and controlled pore sizes [17,18,19]. The latter is primarily constructed using additive manufacturing (AM) processes, which also allow the fabrication of complete implants with smooth or structured surfaces [20,21,22].

AM processes facilitate a diverse variety of scaffold designs, in contrast to classical fabrication techniques, like casting or forging. Scaffolds are fabricated layer by layer from powder particles, melted or sintered at defined areas with a high-energy beam (laser or electron beam) [17,23]. This offers the ability to control of the architecture, and thus the mechanical properties of the scaffolds can be directly driven by the designs, which are virtually limitless.

Nevertheless, the mechanical properties of open-porous scaffolds are often correlated with their porosity, with controversial findings about the validity [24,25,26].

Furthermore, in addition to the mechanical properties, cell biological compatibility also plays an important role in bone ingrowth into the implant material. Open-porous implants made of titanium or its alloys have already demonstrated their ability for osteointegration *in vitro* [23,27,28] and have also been successfully used in *in vivo* studies [18]. Nevertheless, the geometry and size of the pores influence the spreading and proliferation of bone cells [29,30].

In order to assess the suitability of open-porous titanium scaffolds with controlled pore geometry as bone scaffolds, mechanical tests were performed on three different scaffold designs characterized by similar porosities. Stress-strain relationships, as well as mechanical properties (structural modulus, ultimate compressive strength, and ultimate compressive strain), were analyzed.

For *in vitro* testing, human osteoblasts were seeded onto the surfaces of selective laser melting (SLM)-fabricated scaffolds, which had pore geometries similar to the mechanically tested scaffolds, in order to analyze the migration capacity of cells within the scaffold pores. Furthermore, the type I pro‑collagen synthesis ability of the bone cells was determined.

## 2. Materials & Methods

### 2.1. Generating the Open-Porous Scaffolds

**Figure 1:**
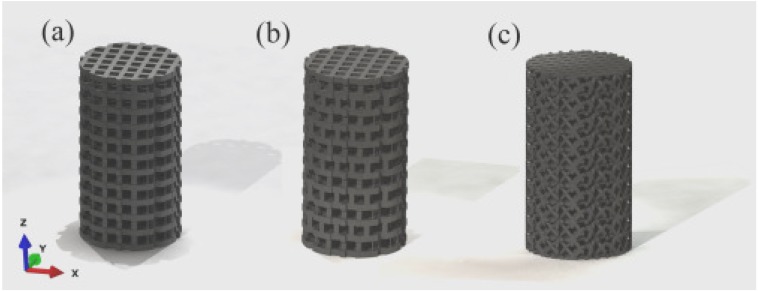
CAD models of the three investigated structures: (**a**) scaffold with rectangular struts aligned in the vertical direction; (**b**) scaffold with shifted strut alignment in *x*–*z* plane; and (**c**) scaffold with diagonally-orientated circular struts.

The scaffold designs were generated using CAD software (SolidWorks 2008; SolidWorks Corporation, Concord, Massachusetts, USA). Three different designs were created (Figure 1), featuring varying structural shapes and strut orientations. The height and diameter of the samples were 14.8 and 4.0 mm, respectively. The first two designs exhibited struts with a rectangular cross-section, orientated vertically. The strut width and height were 400 and 800 µm, respectively. The distance between the two layers was 1.3 mm, and the pore size was 800 × 800 µm. In the case of the first scaffold, the structural shapes in the *x*–*z* and *y*–*z* planes were identical. For the second scaffold, the vertical struts were shifted by half the strut height (*i.e.*, 400 µm) in the *x*–*z* plane. The struts for the third scaffold were orientated diagonally to the vertical axis and exhibited a circular cross-section with a diameter of approximately 300 µm. The distance between the layers was 1.2 mm, and the pore size was approximately 550 × 550 µm.

### 2.2. Fabrication of the Scaffolds

**Figure 2:**
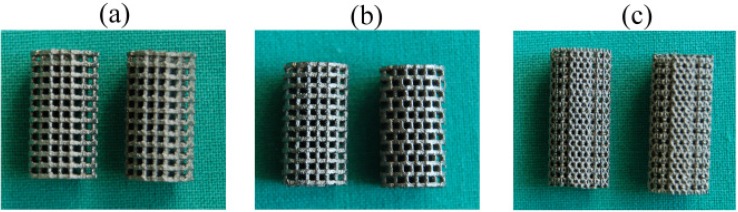
(**a**) Scaffold with identical strut design in *x*–*z* and *y*–*z* plane; (**b**) scaffold with shifted strut orientation; and (**c**) **s** caffold with diagonal struts.

Based on the CAD data, the scaffolds (n = 3 for each design) were fabricated by means of a selective laser melting process (SLM solutions GmbH, Lübeck, Germany) from titanium powder (Ti6Al4V). The manufactured scaffolds are shown in Figure 2.

### 2.3. Calculating Scaffold Porosity

Porosity values for the CAD scaffolds with idealized geometry, and also for the additive manufactured scaffolds (AMS), were calculated according to the following equations:

(1) $$Porosity_{CAD}=\;\left(1-\frac{V_{str}}{V_{cyl}}\right)\;\times \;100\%$$

where *V_str_* is the volume of the CAD scaffold struts and *V_cyl_* is the overall volume enclosed by the outer periphery.

(2) $$Porosity_{AMS}=\;\left(1-\frac{\rho_{sc}}{\rho_{0}}\right)\;\times \;100\%$$

where *ρ_0_* is the density of non-porous Ti6Al4V (4.43 g/cm³) and *ρ_sc_* is the density of the manufactured scaffolds, calculated using the weight and volume of the scaffolds.

### 2.4. Axial Compression Testing

All scaffolds were mechanically tested, in order to determine their mechanical properties. Axial compression tests until mechanical failure were carried out using a universal testing machine (Z50; Zwick Roell, Ulm, Germany) with a traverse velocity of 1.0 mm/min for all scaffolds. Values of applied load and displacement were continuously recorded during testing.

The elastic modulus for each scaffold was calculated, using the applied load and displacement of the testing machine, together with the geometric parameters of the manufactured test samples, according to Equation (3):

(3) $$E_{s}=\frac{F_{R}\cdot l_{0}}{A\cdot \Delta l}$$

where *F_R_* is the applied load, *l_0_* is the initial length, *A* is the initial cross-sectional area, and ∆*l* is the shortening of the scaffold during testing. In addition, ultimate compressive strength and ultimate compressive strain were calculated from the stress-strain relationship.

### 2.5. Cell Seeding on SLM Scaffolds

In order to determine the biological suitability of the SLM-fabricated scaffolds, the migration of human osteoblasts was analyzed. Isolation and cultivation followed the procedure described by Jonitz *et al.* [31].

Scaffolds for *in vitro* testing were made as discs, using the same SLM manufacturing process as the scaffolds used for mechanical testing. These *in vitro* scaffolds were 5 mm in height and 30 mm in diameter, with a rectangular pore size of approximately 700 × 700 µm in all three spatial directions. After cleaning in an ultrasonic bath, scaffolds were sterilized in an autoclave. In order to determine cell proliferation on a scaffold, two discs were put together. As a result, the complete scaffold module was composed of two discs with four different planes (plane 1: superior; planes 2 and 3: intermediate; plane 4: inferior). Human osteoblasts (4 × 10^5^ cells) were then seeded point-wise as a 10 µL cell suspension onto the top surface of this two-piece scaffold, which now had a total height of 10 mm.

### 2.6. In Vitro Characterization

Characterization of the mitochondrial activity of the bone cells and the synthesis of pro-collagen type I was determined using a WST-1 assay and an enzyme-linked immunosorbent assay, respectively. Furthermore, cell viability was analyzed by means of the LIVE/DEAD assay. All procedures are described in detail by Jonitz *et al.* [31].

## 3. Results

### 3.1. Scaffold Porosity

The calculated porosities for the CAD scaffolds and manufactured samples are listed in Table 1. All scaffolds exhibited a porosity of approximately 70%. Deviation between the idealized structure and the corresponding manufactured structure was less than 2%. Moreover, variations within each of the three manufactured structures were less than 1%.

**Table 1:** Porosities of the three scaffold types, calculated from the CAD and for the manufactured samples (AMS), given as means ± (in the case of AMS) standard deviations.

Scaffold	Porosity	
	CAD	AMS
1	70.3%	70.2 ± 0.4%
2	70.3%	71.9 ± 0.2%
3	70.7%	68.7 ± 0.2%

### 3.2. Mechanical Behavior

**Figure 3:**
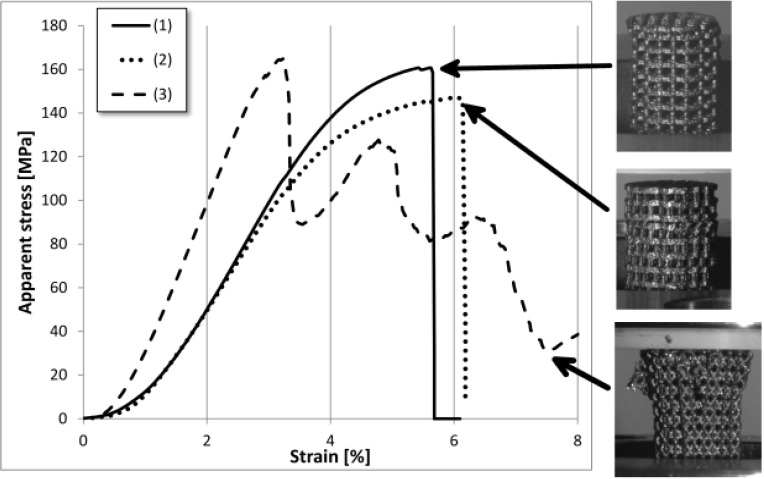
Engineering stress-strain relationships for the three types of manufactured scaffold. One graph is plotted for each type of scaffold, as an example: (**1**) rectangular struts; (**2**) rectangular struts with shifted strut alignment; and (**3**) diagonally-orientated struts. Photographs of the mechanical failure for each type of scaffold are shown to the right.

Apparent stress-strain relationships for the scaffolds were calculated on the basis of the nominal cross-sectional areas of the scaffolds (Figure 3). Consequently, apparent stress did not reflect the true stress within the scaffold (*i.e.*, the struts). For each type of scaffold, only one graph has been plotted as an example.

Both types of scaffold with rectangular-shaped struts (Figure 3, lines 1 and 2) exhibited an elastic response with a small region of yielding, followed by a sudden decrease at approximately 6% strain due to the failure of the vertical struts within a single layer. These layers were completely separated from the scaffold, shifted slightly sideways and could be easily removed after unloading. Nevertheless, the remaining scaffolds did not present any indication of plastic deformation.

The third type of scaffold did not exhibit any plastic yielding. Instead, failure of the scaffold occurred stepwise. After each failure the stress increased again. The scaffold displayed disruption of the structural framework along the vertical axis. Furthermore, small parts of the material broke away. Subsequently, damage occurred within the entire scaffold during testing.

### 3.3. Mechanical Properties

The mechanical properties of the scaffolds (*i.e.*, elastic modulus, ultimate compressive strength, and ultimate compressive strain) were derived from the stress-strain relationships. Elastic modulus was calculated from the slope of the elastic response. Ultimate compressive strength was defined as the maximum stress prior to failure. Ultimate compressive strain was the strain corresponding to the ultimate compressive strength. The resultant values are listed in Table 2.

**Table 2:** Mechanical properties of the three scaffold designs, given as means ± standard deviations.

Scaffold design	Elastic modulus (GPa)	Ultimate compressive strength (MPa)	Ultimate compressive strain (%)
**1**	5.1 ± 0.3	155 ± 7	5.22 ± 0.34
**2**	3.7 ± 0.2	145 ± 2	6.70 ± 0.56
**3**	6.7 ± 0.3	164 ± 6	3.45 ± 0.50

### 3.4. In Vitro Properties

**Figure 4:**
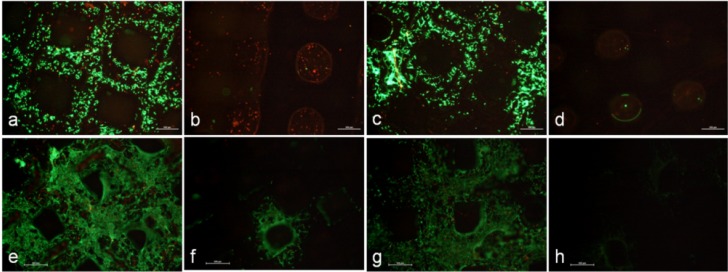
Viability of human osteoblasts on the various planes of the 3D Ti6Al4V scaffold on day 1 (**a**–**d**) and day 8 (**e**–**h**) of cultivation under static culture conditions (n ≥ 3; living cells = green; dead cells = red; scale bar = 500 µm; (**a**), (**e**): plane 1; (**b**), (**f**): plane 2; (**c**), (**g**): plane 3; and (**d**), (**h**): plane 4).

To analyze the viability of human osteoblasts within the scaffold, cells were seeded onto the superior plane 1. One day after seeding, a lot of cells were observed on planes 1 and 3, whereas no living cells were detected on planes 2 and 4. After 8 days of cultivation, a lot of viable cells were detectable on planes 1–3. These cells also formed numerous cell connections, which resulted in a densely populated surface on both planes. It was also striking that a lot of dead cells could be determined within unpopulated areas. In contrast, there were only a few cells on the intermediate plane 2, and no cells were visible on plane 4 (Figure 4).

**Figure 5:**
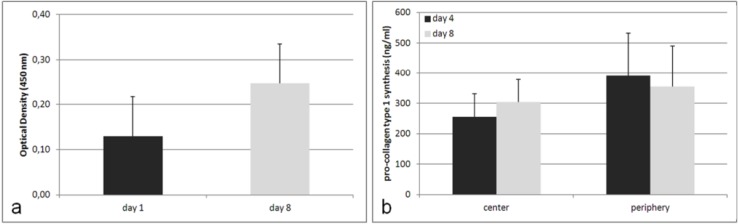
(**a**) Metabolic activity of human osteoblasts seeded onto the 3D Ti6Al4V scaffold, after 8 days of cultivation (n = 3); and (**b**) synthesis of pro-collagen type I from human osteoblasts seeded onto the 3D Ti6Al4V scaffold. Supernatants were collected after 4 and 8 days of cultivation and analyzed using ELISA (n = 3). Data are given as means ± standard deviations.

Additionally, the experimental setup was disassembled after days 1 and 8 of cultivation, in order to perform WST-1 assays. On both occasions, metabolically-active cells were determined, whereby the metabolic activity was observed to increase two-fold during the incubation time (Figure 5a). To obtain information on the synthesis of the ECM components, medium from the center and periphery of the scaffold was analyzed after 4 and 8 days of cultivation, during which time the synthesis rate of type I pro-collagen increased from 304 to 355 ng/mL at the periphery, and from 255 to 391 ng/mL in the center (Figure 5b).

## 4. Discussion

The challenge for a metallic implants is to provide sufficient mechanical stability, and also adaptation of mechanical properties to the surrounding tissue, in order to prevent bone loss due to stress shielding [10,11,32]. Adaptation to the mechanical properties of the bone must be considered, especially when dealing with open-porous scaffolds or surface coatings, where the bone is supposed to penetrate into the scaffold [33].

The use of additive manufacturing processes, such as laser or electron beam melting, offers the possibility to fabricate open-porous bone scaffolds, as well as non-porous implants, in a wide range of shapes and structures. Furthermore, by gaining full control of the shape and geometric composition, any desired mechanical properties can be directly controlled by the structural design.

Nevertheless, the mechanical properties of additive manufactured samples are similar to those manufactured conventionally. Koike *et al.* [17] compared the mechanical properties of tensile test samples fabricated by selective laser and electron beam melting with cast and wrought samples. Their results showed comparable yield and tensile strength values for the four different samples. Laser beam melted and wrought specimens had similar strengths, whereas electron beam melted specimens had slightly less strength. Furthermore, the yield strength of the cast samples was lowest of all.

In the present study, the mechanical properties of open-porous scaffolds, with three different designs, were determined. Porosity calculations revealed similar values, of approximately 70%, for all three scaffold designs. Although geometric structure and strut orientation differed between the three designs, ultimate compressive strengths were similar for all scaffolds, varying between 145 and 164 MPa. These values are within the range of 140–220 MPa reported for human cortical bone, determined by experimental testing [34,35].

In contrast, greater differences were observed between the three designs in terms of elastic modulus, which varied between 3.7 and 6.7 GPa. These findings support the argument that the mechanical properties of scaffolds may not only be influenced by porosity. Since only three different structural designs were examined in the present study, our conclusions may not be significant, but nevertheless support previous findings published in the literature.

Murr *et al*. [20] fabricated cubic scaffolds of varying density using the electron beam melting (EBM) process. They found different mechanical property values for similar porosity values (82%). Scaffolds with strut thicknesses of 1.0 and 1.2 mm exhibited elastic moduli of 1.5 and 0.9 GPa, respectively.

Parthasarathy *et al.* [24] also used EBM to fabricate open-porous scaffolds with porosities ranging from 50% to 70%. All scaffold struts exhibited a rectangular cross-section, with two different strut thicknesses (450 and 800 µm). In general, the scaffolds demonstrated a decrease in mechanical properties with increasing porosity. In contrast, significantly different mechanical property values were observed, despite the fact that two of the scaffolds had similar porosities (approximately 50%). The resultant compressive stiffnesses were 2.9 and 0.6 GPa for scaffolds with strut thicknesses of 800 and 450 µm, respectively.

The elastic modulus results of Murr *et al*. [20] (2.7 GPa for 72% porosity) and Parthasarathy *et al.* [24] (2.1 GPa for 70% porosity) were lower than the values obtained during the current study (5.1 GPa for 70% porosity). These differences can be explained by variations in the fabrication methods (*i.e.*, laser and electron beam melting). Samples fabricated by EBM were generally characterized by rougher surfaces than those made using SLM [17], and thus exhibited lower mechanical properties [36]. Nevertheless, all elastic modulus results were lower than the values for human cortical bone, which fall in the range 15–20 GPa [34,35].

Regarding the biocompatibility of the examined material *in vitro*, the results of the current study suggest that human osteoblasts could survive on porous titanium scaffolds in a static cell culture. Furthermore, the synthesis of pro-collagen type I was not only sustained during the incubation period, it clearly increased. The scaffold macropores were settled by cells, although the pore size prevented an overgrowing of cells. Other studies also indicated the proliferation of human osteoblasts on scaffolds made of titanium [9,28].

Hollander *et al.* [28] seeded human osteoblasts onto the surface of additive manufactured titanium scaffolds with a regular, circular pore diameter of approximately 500–1000 µm. Their results showed proliferation and survival of the cells after 14 days of cultivation. Furthermore, the pores of the scaffolds filled with cells, which had grown along the pore rims.

Mueller *et al.* [9] seeded human osteoblasts onto foam-like open-porous titanium scaffolds with pore diameters between 100 and 750 µm. Under static culture conditions, and in a perfused system, they demonstrated that human osteoblasts grew through the interconnected pores of the metal foam, and expressed an osteoblast-like phenotype.

Cell proliferation was also observed during the present study, which implies bone ingrowth into titanium implants in a biological environment, as described by Mangano *et al.* [23].

It should be noted that the cell investigations presented here were performed on only one geometric scaffold type. It is possible that cell behavior could vary between pores of different geometric shape. Nevertheless, methodical investigations of cell proliferation in different pore sizes are rare.

Frosch *et al.* [37] determined the effect of different diameters of cylindrical titanium channels on human osteoblast cell proliferation. Pores with diameters of between 300 and 1000 µm were drilled into a titanium block, such that there were no interconnections. The experiments indicated that channel diameter had no influence on collagen type I production. Furthermore, the highest osteogenic differentiation was found in 600 µm pores, whereas the highest cell density was in 300 µm pores.

In summary, the current study demonstrated the influence of three different scaffold designs on mechanical properties, providing an open-porous design with adequate pore geometry [9]. Furthermore, it was shown that a low elastic modulus can stimulate new bone formation, due to mechanical stimulus by physiological load application, and avoid stress shielding caused by high stiffness gradients between bones and implants [10,11]. Nevertheless, in instances of large segmental defects in long bones, initial stabilization of open-porous scaffolds should be supported by osteosynthesis systems, such as intramedullary nails, plates or by external fixation. Consequently, stress distribution within the bone-implant interface in such a complex situation should be further analyzed, in order to obtain a realistic prediction of the situation *in vivo*. The results of the *in vitro* testing indicated a high degree of human osteoblast (which are very sensitive to artificial materials) cell proliferation on the titanium surface.

## 5. Conclusions

Using additive manufacturing process SLM, the design diversity and adaptation to nearly any desired target value can be implemented to open-porous bone scaffolds. Furthermore, the mechanical load on the bone can be controlled and consequently the stimulation for bone regeneration as well as stress shielding due to the optimized material properties. It is assumed that the proliferation and survival of cells onto the titanium surface would lead to complete bone ingrowth into the implant under *in vivo* conditions.

## Supplementary Materials

Supplementary File 1

## References

[1] De Coster T.A., Gehlert R.J., Mikola E.A., Pirela-Cruz M.A. (2004). Management of posttraumatic segmental bone defects. J. Am. Acad. Orthop. Surg..

[2] Attias N., Lehman R.E., Bodell L.S., Lindsey R.W. (2005). Surgical management of a long segmental defect of the humerus using a cylindrical titanium mesh cage and plates: A case report. J. Orthop. Trauma.

[3] Schieker M., Mutschler W. (2006). Bridging posttraumatic bony defects. Established and new methods. Unfallchirurg.

[4] Dumont C.E., Exner U.G. (2009). Reconstruction of large diaphyseal defects of the femur and the tibia with autologous bone. Eur. J. Trauma Emerg. Surg..

[5] Niedhart C., Pingsmann A., Jurgens C., Marr A., Blatt R., Niethard F.U. (2003). Complications after harvesting of autologous bone from the ventral and dorsal iliac crest—A prospective, controlled study. Z. Orthop. Grenzgeb..

[6] Younger E.M., Chapman M.W. (1989). Morbidity at bone graft donor sites. J. Orthop. Trauma.

[7] Hutmacher D.W., Schantz J.T., Lam C.X., Tan K.C., Lim T.C. (2007). State of the art and future directions of scaffold-based bone engineering from a biomaterials perspective. J. Tissue Eng. Regen. Med..

[8] Reichert J.C., Wullschleger M.E., Cipitria A., Lienau J., Cheng T.K., Schutz M.A., Duda G.N., Noth U., Eulert J., Hutmacher D.W. (2011). Custom-made composite scaffolds for segmental defect repair in long bones. Int. Orthop. 201.

[9] Mueller U., Imwinkelried T., Horst M., Sievers M., Graf-Hausner U. (2006). Do human osteoblasts grow into open-porous titanium?. Eur. Cells Mater..

[10] Antonialli A.I.S., Bolfarini C. (2011). Numerical evaluation of reduction of stress shielding in laser coated hip prostheses. Mater. Res..

[11] Niinomi M., Nakai M. (2011). Titanium-based biomaterials for preventing stress shielding between implant devices and bone. Int. J. Biomater..

[12] Li J.P., Wijn J.R., van Blitterswijk C.A., de Groot K. (2007). Comparison of porous Ti6Al4V made by sponge replication and directly 3D fiber deposition and cancellous bone. Key Eng. Mater..

[13] Karageorgiou V., Kaplan D. (2005). Porosity of 3D biomaterial scaffolds and osteogenesis. Biomaterials.

[14] Ryan G., Pandit A., Apatsidis D.P. (2006). Fabrication methods of porous metals for use in orthopaedic applications. Biomaterials.

[15] Takemoto M., Fujibayashi S., Neo M., Suzuki J., Kokubo T., Nakamura T. (2005). Mechanical properties and osteoconductivity of porous bioactive titanium. Biomaterials.

[16] Spoerke E.D., Murray N.G., Li H., Brinson L.C., Dunand D.C., Stupp S.I. (2005). A bioactive titanium foam scaffold for bone repair. Acta Biomater..

[17] Koike M., Greer P., Owen K., Lilly G., Murr L.E., Gaytan S.M., Martinez E., Okabe T. (2011). Evaluation of titanium alloys fabricated using rapid prototyping technologies—Electron beam melting and laser beam melting. Materials.

[18] Ponader S., von Wilmowsky C., Widenmayer M., Lutz R., Heinl P., Koerner C., Singer R.F., Nkenke E., Neukam F.W., Schlegel K.A. (2010). *In vivo* performance of selective electron beam-melted Ti-6Al-4V structures. J. Biomed. Mater. Res. A.

[19] Li J.P., Habibovic P., van den Doel M., Wilson C.E., de Wijn J.R., van Blitterswijk C.A., de Groot K. (2007). Bone ingrowth in porous titanium implants produced by 3D fiber deposition. Biomaterials.

[20] Murr L.E., Gaytan S.M., Medina F., Lopez H., Martinez E., Machado B.I., Hernandez D.H., Martinez L., Lopez M.I., Wicker R.B., Bracke J. (2010). Next-generation biomedical implants using additive manufacturing of complex, cellular and functional mesh arrays. Phil. Trans. Roy. Soc. A Math. Phys. Eng. Sci..

[21] Harrysson O.L.A., CansiZoglu O., Marcellin-Little D.J., Cormier D.R., West H.A. (2008). Direct metal fabrication of titanium implants with tailored materials and mechanical properties using electron beam melting technology. Mater. Sci. Eng. C Biomim. Supramol. Syst..

[22] Chahine G., Koike M., Okabe T., Smith P., Kovacevic R. (2008). The design and production of Ti-6Al-4V ELI customized dental implants. JOM.

[23] Mangano C., de Rosa A., Desiderio V., d'Aquino R., Piattelli A., de Francesco F., Tirino V., Mangano F., Papaccio G. (2010). The osteoblastic differentiation of dental pulp stem cells and bone formation on different titanium surface textures. Biomaterials.

[24] Parthasarathy J., Starly B., Raman S., Christensen A. (2010). Mechanical evaluation of porous titanium (Ti6Al4V) structures with electron beam melting (EBM). J. Mech. Behav. Biomed. Mater..

[25] Gibson L.J., Ashby M.F. (1982). mechanics of three-dimensional cellular materials. Proc. R Soc. Lond. Ser. A.

[26] Li X., Wang C.T., Zhang W.G., Li Y.C. (2010). Fabrication and compressive properties of Ti6Al4V implant with honeycomb-like structure for biomedical applications. Rapid Prototyping J..

[27] Fassina L., Saino E., Visai L., Magenes G. Electromagnetically enhanced coating of a sintered titanium grid with human SAOS-2 osteoblasts and extracellular matrix. Proceedings of 30th Annual International Conference of the IEEE.

[28] Hollander D.A., von Walter M., Wirtz T., Sellei R., Schmidt-Rohlfing B., Paar O., Erli H.J. (2006). Structural, mechanical and in vitro characterization of individually structured Ti-6Al-4V produced by direct laser forming. Biomaterials.

[29] Frosch K.H., Barvencik F., Lohmann C.H., Viereck V., Siggelkow H., Breme J., Dresing K., Sturmer K.M. (2002). Migration, matrix production and lamellar bone formation of human osteoblast-like cells in porous titanium implants. Cells Tissues Organs.

[30] Warnke P.H., Douglas T., Wollny P., Sherry E., Steiner M., Galonska S., Becker S.T., Springer I.N., Wiltfang J., Sivananthan S. (2009). Rapid prototyping: Porous titanium alloy scaffolds produced by selective laser melting for bone tissue engineering. Tissue Eng. Part C Methods.

[31] Jonitz A., Wieding J., Lochner K., Cornelsen M., Seitz H., Hansmann D., Bader R. (2011). Migration capacity and viability of human primary osteoblasts in synthetic three-dimensional bone scaffolds made of tricalciumphosphate. Materials.

[32] Merle C., Streit M.R., Volz C., Pritsch M., Gotterbarm T., Aldinger P.R. (2011). Bone remodeling around stable uncemented titanium stems during the second decade after total hip arthroplasty: A DXA study at 12 and 17 years. Osteoporos. Int..

[33] Suzuki Y., Nomura N., Hanada S., Kamakura S., Anada T., Fuji T., Honda Y., Masuda T., Sasaki K., Kokubun S., Suzuki O. (2007). Osteoconductivity of porous titanium having young’s modulus similar to bone and surface modification by OCP. Key Eng. Mater..

[34] Ohman C., Baleani M., Pani C., Taddei F., Alberghini M., Viceconti M., Manfrini M. (2011). Compressive behaviour of child and adult cortical bone. Bone.

[35] Grimal Q., Haupert S., Mitton D., Vastel L., Laugier P. (2009). Assessment of cortical bone elasticity and strength: Mechanical testing and ultrasound provide complementary data. Med. Eng. Phys..

[36] Cahill S., Lohfeld S., McHugh P.E. (2009). Finite element predictions compared to experimental results for the effective modulus of bone tissue engineering scaffolds fabricated by selective laser sintering. J. Mater. Sci. Mater. Med..

[37] Frosch K.H., Barvencik F., Viereck V., Lohmann C.H., Dresing K., Breme J., Brunner E., Sturmer K.M. (2004). Growth behavior, matrix production, and gene expression of human osteoblasts in defined cylindrical titanium channels. J. Biomed. Mater. Res. A.

