# Supplementary figures and images for: The Effect of Structural Design on Mechanical Properties and Cellular Response of Additive Manufactured Titanium Scaffolds
Source: Materials (Basel). 2012 Aug 10;5(8):1336–47. doi: 10.3390/ma5081336 (PMC5448937; doi:10.3390/ma5081336)

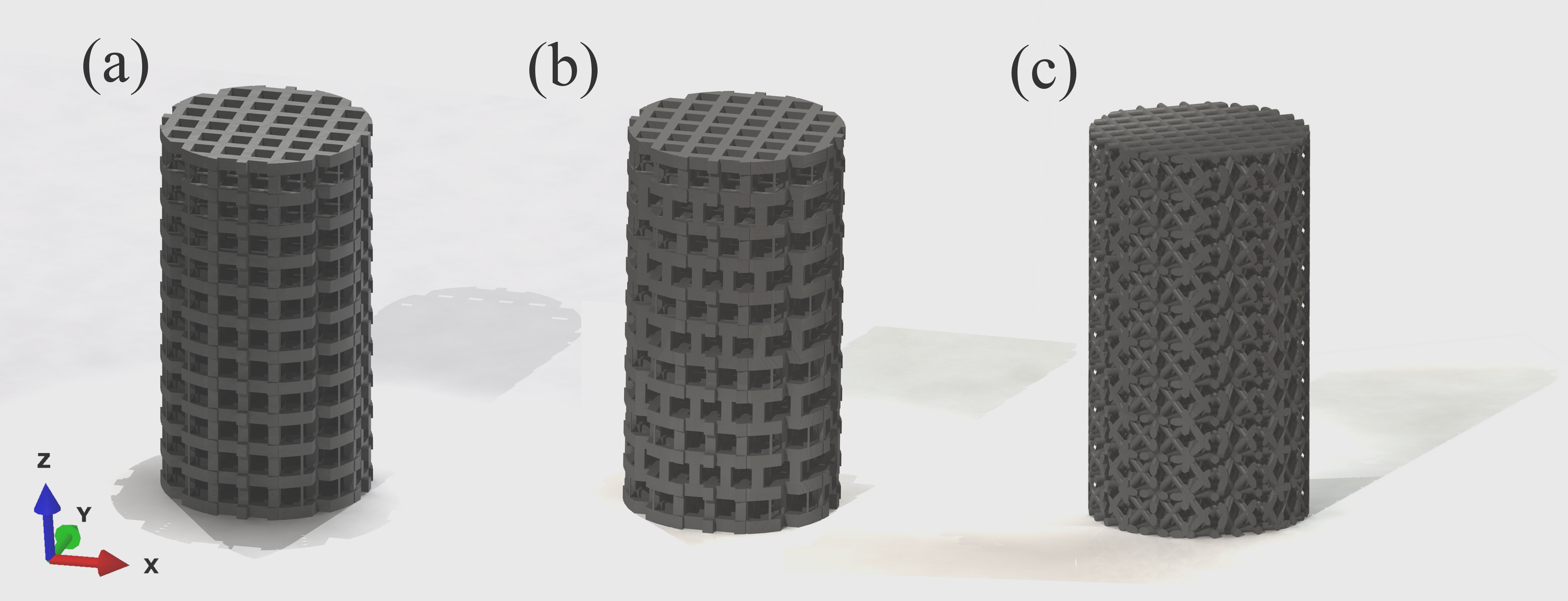

Supplement: Supplementary File 1 [file materials-05-01336-s001.zip › Fig1.tif]

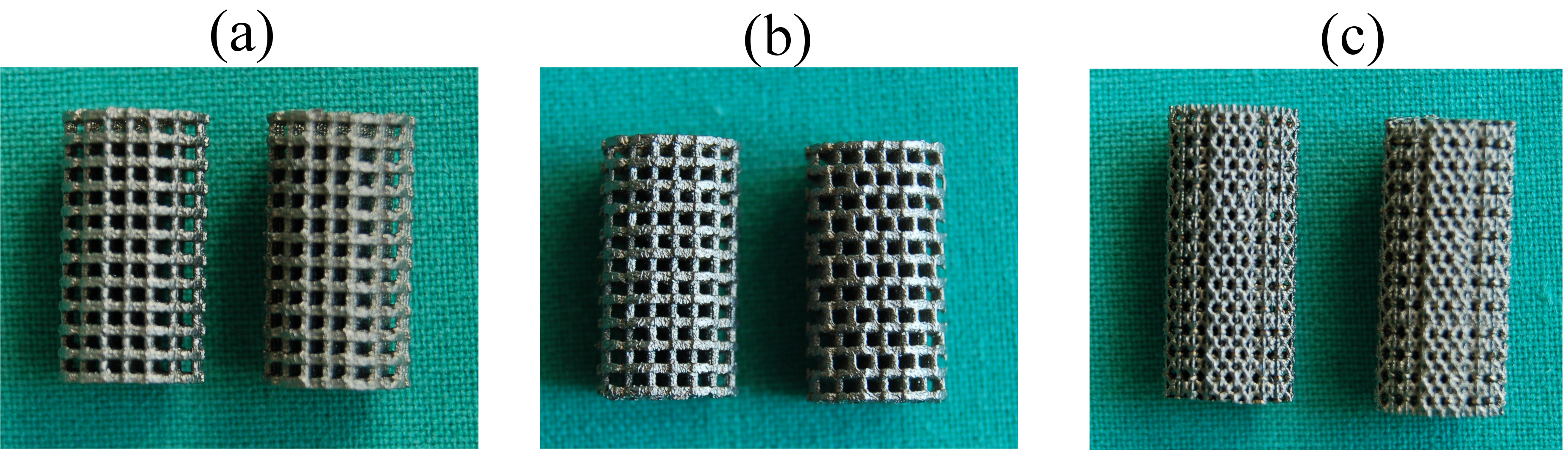

Supplement: Supplementary File 1 [file materials-05-01336-s001.zip › Fig2.tif]

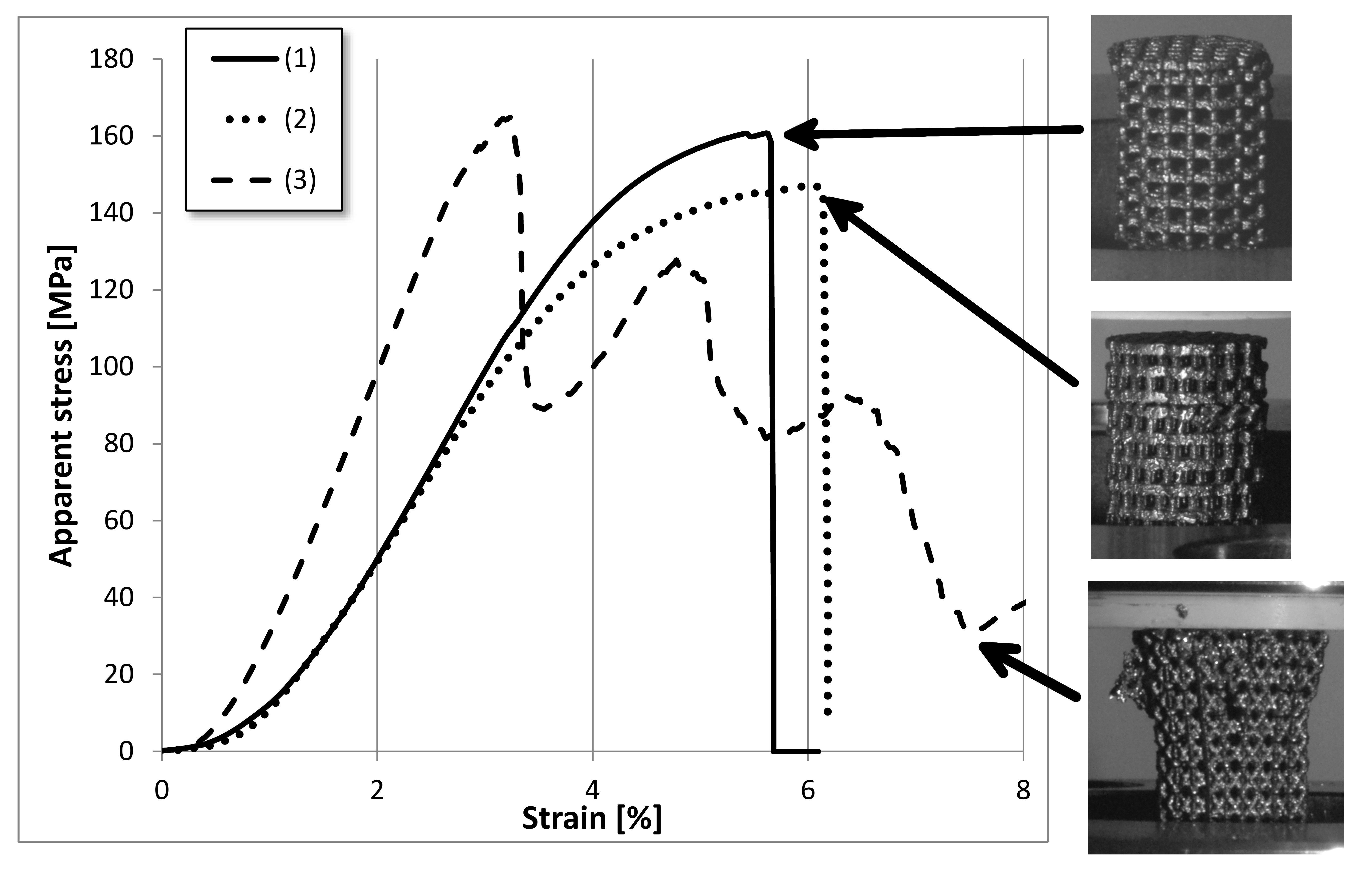

Supplement: Supplementary File 1 [file materials-05-01336-s001.zip › Fig3.tif]

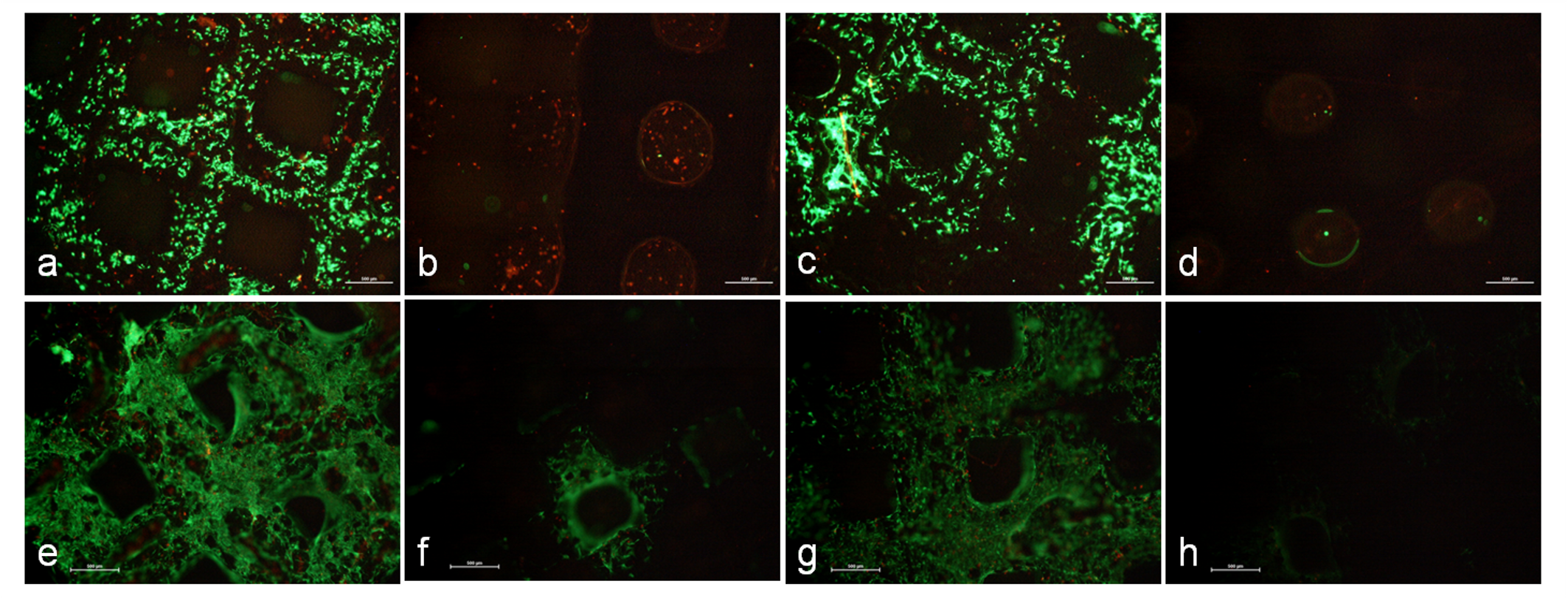

Supplement: Supplementary File 1 [file materials-05-01336-s001.zip › Fig4.tif]

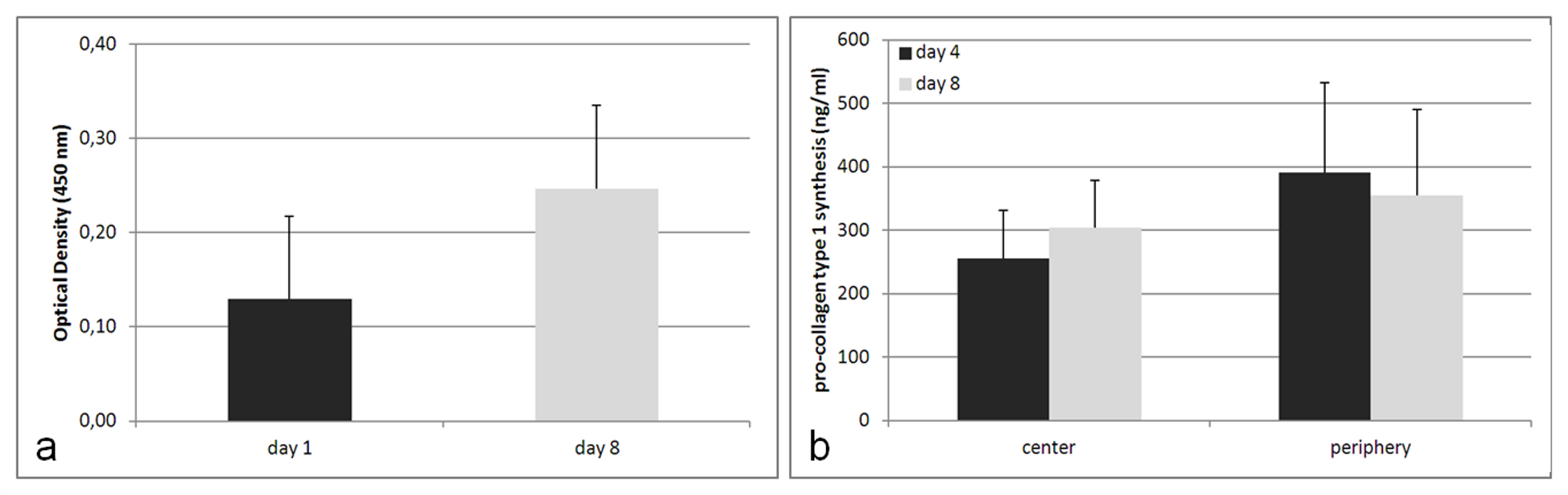

Supplement: Supplementary File 1 [file materials-05-01336-s001.zip › Fig5.tif]
